# Pvr and Pvf2 Are Essential for Valve Cell Differentiation in the Larval *Drosophila* Heart

**DOI:** 10.1002/dvg.70053

**Published:** 2026-05-09

**Authors:** Heiko Meyer, Christian Meyer, Bettina Priess, Achim Paululat

**Affiliations:** ^1^ Department of Biology/Chemistry, Zoology & Developmental Biology Osnabrück University Osnabrück Germany; ^2^ Center of Cellular Nanoanalytics (CellNanOs), Osnabrück University Osnabrück Germany

**Keywords:** heart function, Pvf1, Pvf2, Pvf3, Pvr, valve cells

## Abstract

The directionality of blood flow is regulated by heart valves, among other things. While the heart valves in vertebrates are multicellular and complex, the valve in the *Drosophila* heart consists of exactly two highly specialized cells. They arise during early larval development from two cardiomyocytes, those that form the boundary between the aorta and the posterior ventricle. Here, we show that the conserved PDGF signaling pathway is involved in the determination of heart valve cells. RNAi‐mediated knockdown of the Pvr receptor and one of its ligands, Pvf2, leads to an inhibition of valve cell differentiation. In contrast, the simultaneous expression of the Pvr and Pvf2 in the entire heart tube leads to the formation of additional heart valve cells in ectopic positions. A single expression of the receptor or the ligand does not lead to heart valve formation.

## 1 Introduction

**FIGURE 1:**
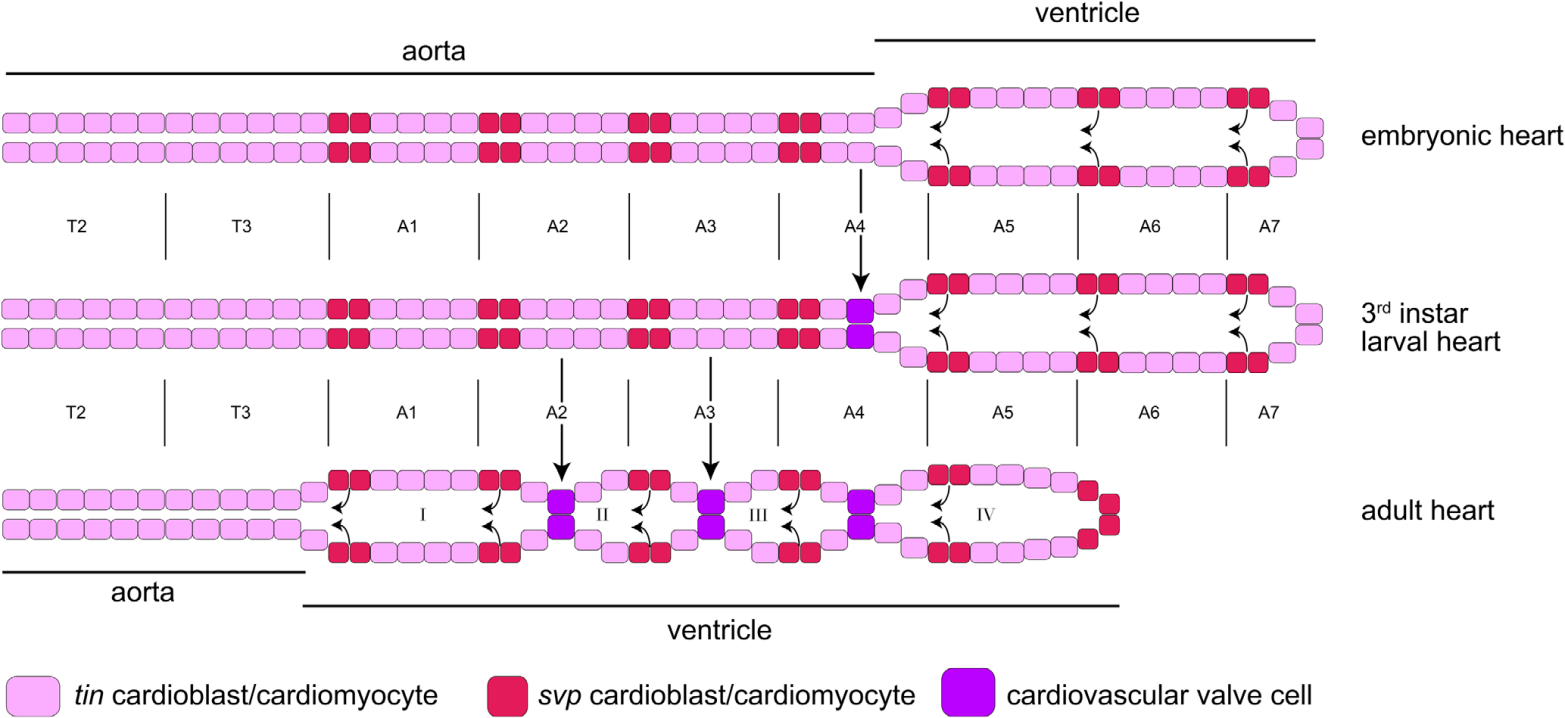
Heart development from the embryo to the adult stage. The scheme shows the heart tube in embryos (upper panel), in third‐instar larvae (middle panel), and an adult fly (lower panel). Only cardiac muscle cells (pink), ostia cells (red), and heart valve cells (purple) are shown. In the larva, a single pair of heart valve cells separates the anterior aorta from the posterior large‐lumen ventricle. In adult animals, three pairs of heart valve cells divide the heart tube into four distinct sections. Arrows mark the normal direction of hemolymph flow.

Insects possess an open circulatory system where hemolymph (insect blood) circulates within the body cavity, driven by a tubular heart, also called the dorsal vessel. The heart tube comprises several cell types with specific functions: contractile cardiomyocytes, gate‐forming ostial cells that permit hemolymph to enter the heart, and intracardiac valve cells that ensure unidirectional hemolymph flow, a function analogous to the valves of the human heart (Figure 1). The differentiation and function of the *Drosophila* heart and most of its cell types have been well described in numerous publications, including valve cells, which have received increasing attention in recent years (Lammers et al. 2017; Lehmacher et al. 2012; Meyer, Bataille, et al. 2023; Meyer et al. 2022; Meyer, Drechsler, et al. 2023; Meyer and Paululat 2025; Zeitouni et al. 2007). This was possible because valve cell‐specific markers and mutants have been identified, enabling not only genetic studies but also live imaging and hemolymph flow analyses, prerequisites for functional studies. The primary function of the cardiac valves is to regulate directional hemolymph flow within the heart tube and to prevent backflow, thereby optimizing overall cardiac performance (Meyer and Paululat 2025). The valve cells operate through dynamic, oscillating changes in shape. During diastole (heart filling), the cells adopt a spherical shape, thereby sealing the lumen between the heart chamber and the aorta and facilitating efficient intake of hemolymph from the abdominal cavity. During systole (heart contraction), the cells deform and flatten, opening the exit of the ventricle to allow the accumulated hemolymph to be pumped anteriorly into the aorta. The recurring switch between spherical and flattened cell shapes is enabled by the specific histology of the valve cells, particularly by the presence of huge intracellular membrane compartments, which we named valvosomes. The histology of the valve cells has previously been described in detail (Lammers et al. 2017; Lehmacher et al. 2012). The mechanical action of the valve cells is further supported by hemolymph pressure and the unique crisscross orientation of myofibrils within the valve cells, which enables the rapid shape changes necessary for opening and closing the heart channel during each beat (Meyer et al. 2022; Meyer, Drechsler, et al. 2023). The physiological importance of functional heart valves in *Drosophila* has recently been confirmed via genetic manipulation experiments (Meyer and Paululat 2025). Flies with malformed or nonfunctional valve cells exhibit a measurable reduction in heart pumping capacity and decreased overall mobility and fitness. This functional impairment underscores the essential role of valves even in the low‐pressure, open circulatory system of insects.

### 1.1 PDGF‐VEGF Signaling Is Required for Larval Valve Formation

The mechanisms that trigger valve cell differentiation in the fly heart remain largely unknown. Tang et al. (2013, 2014) found that the transcriptional adapter protein Pygopus, a component of the Wnt signaling pathway, plays a role in differentiation and orientation of the myofibrils in valve cells. Downregulation of Pygopus in adult flies results in smaller valve cells. Recently, we have shown that Tail‐up (Tup) plays a role in myofibril orientation in valve cells (Meyer, Bataille, et al. 2023). In a gene expression profiling analysis of the adult heart, Zeitouni et al. (2007) found that the PDGF‐VEGF pathway plays an essential role in valve cell formation in the adult fly. They found that the PDGF‐ and VEGF‐receptor related (Pvr) is expressed in valve cells from 33 h APF (after puparium formation) onward. This coincides with the transient expression of one of its ligands, PDGF‐ and VEGF‐related factor 2 (Pvf2) (from 27 to 42 h APF). Indeed, downregulation of Pvr function with Pvr^DN^ repressed valve formation in 20% of the cardiac tubes analyzed (*n* = 30), while ectopic expression of the activated Pvr protein (Pvr^lambda^) induced ectopic valve formation in 45% of the cases (*n* = 20) (Zeitouni et al. 2007). Zeitouni and colleagues studied heart formation during metamorphosis, focusing on the formation of the adult heart, which is subdivided into four chambers by three valves (Figure 1).

However, the larval heart is different with respect to its heart chambers and the number of intracardiac valves. During embryogenesis and early larval development, two of the 104 cardiomyocytes that form the heart tube differentiate into cardiac valve cells that form an oscillating closure mechanism for the heart tube, owing to their characteristic subcellular architecture. This single pair of valve cells differentiates at the junction of the posterior heart chamber (ventricle) and the anterior aorta (Figure 1). During metamorphosis, the heart undergoes significant remodeling. However, the single larval valve is maintained, and two additional valves develop from existing cardiomyocytes, resulting in a four‐chambered adult heart with three functional valves.

Here, we present our findings on how the single larval cardiac valve differentiates. We found that the PDGF‐ and VEGF‐receptor related (Pvr) signaling pathway is essential for initiating valve cell differentiation in the larval heart. As Zeitouni and colleagues have shown, the same signaling pathway plays a critical role in the determination of adult heart valves (Zeitouni et al. 2007). Interestingly, in vertebrates, PDGF signaling directs cardiomyocyte movement toward the midline during heart tube assembly and thus is essential for correct heart formation. Mutation of PDGFRα disrupts heart tube assembly in both zebrafish and mouse (Bloomekatz et al. 2017). Moreover, PDGFRα is crucial for stabilizing the valve endocardium after the endothelial‐to‐mesenchymal transition (Van den Akker et al. 2008). Although the histology and anatomy of insect and vertebrate hearts are not directly comparable, the same conserved molecular signaling pathways appear to be involved in the formation of essential heart structures.

## 2 Results

### 2.1 Larval Expression of Pvr and Its Ligands

**FIGURE 2:**
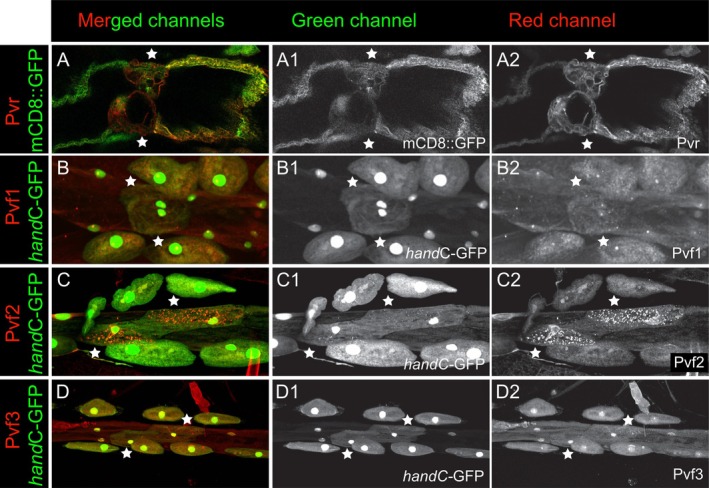
Characterization of Pvr, Pvf1, Pvf2, and Pvf3 expression in the third instar larval heart by immunofluorescence analysis. (A, A1 and A2) Pvr (red) is detected in the membrane (mCD8GFP, green) of larval valve cells (asterisks) and the posterior adjacent cells in the ventricle. (B, B1 and B2) Pvf1 (red) is not expressed in the valve cells (asterisks) and not in the heart of third instar larvae at detectable levels. (C, C1 and C2) A distinct signal of Pvf2 (red) is observed in a punctate manner in the cytoplasm of valve cells (asterisks). (D, D1 and D2) Pvf3 (red) is absent in the valve cells (asterisks) as well as in the larval heart.

Stimulated by the findings of Zeitouni et al. (2007), we investigated the expression patterns of the receptor Pvr and its ligands during larval valve cell development. Since three Pvr ligands are known in *Drosophila*, all three (Pvf1, Pvf2, and Pvf3) were investigated by antibody staining for the respective proteins and by counterstaining for *handC*‐GFP, which we crossed in to identify the heart tube and valve cells (Figure 2). Pvr expression was already observed in pupal valves and could now be confirmed for larval valve cells (Figure 2A). Pvr associates with the cell membrane, as expected for a cell surface receptor. Furthermore, Pvr was present in the neighboring cardiomyocytes of the heart tube next to the valve cells. Expression of the ligand Pvf1 could not be identified in the heart tube, nor in the cardiovascular valve cells of third instar larvae (Figure 2B).

In contrast, Pvf2 was highly expressed in valve cells of third instar larvae (Figure 2C) and localized to distinct punctate intracellular compartments, likely secretory vesicles. Such staining is expected for a secreted ligand. Immuno‐staining against Pvf3 resulted only in a background signal, thus no relevant Pvf3 expression could be observed in the heart of third instar larvae (Figure 2D). Our results showed that the receptor Pvr and its ligand Pvf2 co‐localize in the cardiovascular valve cells of third instar larvae.

### 2.2 The Embryonic Heart Lacks Valve Cells as Well as a Specific Expression of Pvr or of Any of Its Ligands

**FIGURE 3:**
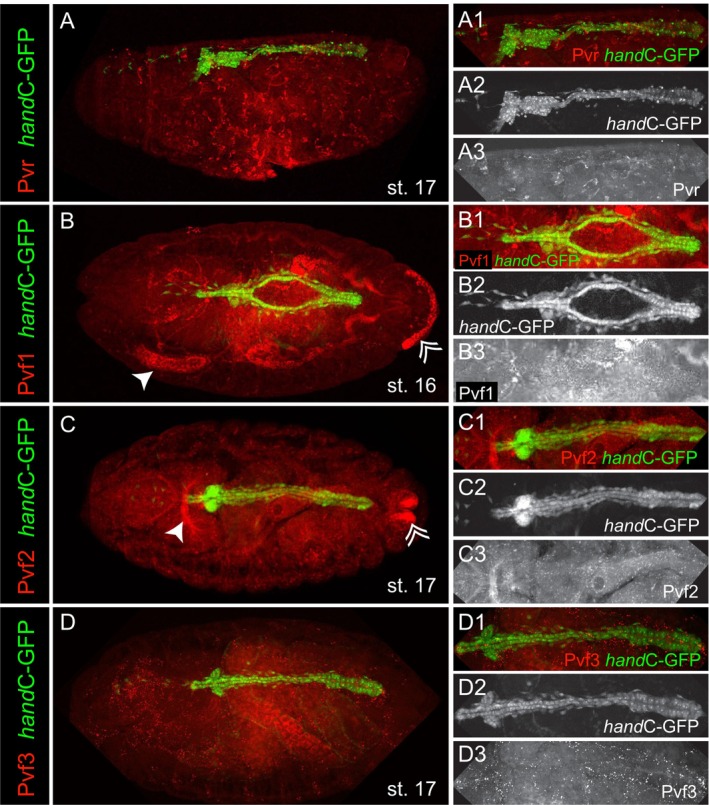
Antibody staining of embryos of the reporter line *handC*‐GFP, stained for GFP (green) and the receptor (Pvr, red) or the individual ligands (Pvf1, Pvf2, Pvf3, red). (A) Pvr is found in hemocytes in Stage 17 embryos. (A1–A3) Within the heart tube, Pvr signaling is absent. (B) Expression of the ligand Pvf1 is present in the salivary glands (arrow) and the anal plate primordium (double arrow) in the embryo (Stage 16). (B1–B3) Pvf1 is not present in the dorsal vessel. (C) Pvf2 is present in the proventriculus (arrow) and the tip of the posterior spiracles (double arrow). (C1–C3) Pvf2 is absent in the embryonic heart tube. (D) Staining of Pvf3 did not result in a specific signal. (D1–D3) Expression of Pvf3 was not found in the dorsal vessel of a stage 17 embryo.

The expression of Pvr and Pvf2 in third instar larval valve cells suggests that both factors may play a role in valve cell differentiation. However, our previous studies on the ultrastructure of heart cells at early developmental stages indicated that valve cell formation starts after embryogenesis. The first sign of valve cell differentiation, based on ultrastructural criteria, is observed in late first‐instar larvae (Lammers et al. 2017). We examined the expression of Pvr and its ligands, Pvf1, Pvf2, and Pvf3, in stage 16 embryos (Figure 3) and found that neither Pvr nor any of its potential ligands was specifically expressed at detectable levels in cardiac tissue.

In detail, Pvr is expected to be expressed in embryonic hemocytes, which distribute throughout the embryos as migratory cells (Cho et al. 2002; Heino et al. 2001). Indeed, we observe patchy Pvr expression in single cells that appear randomly distributed in the embryo, which may correspond to hematopoietic cells in late embryos. Thus, our stainings confirm previous observations (Figure 3A). However, no Pvr expression was visible in the embryonic heart (Figure 3A). The ligand Pvf1 is reported to be strongly expressed in the developing salivary glands of the embryo, a finding confirmed by our immunostainings (Figure 3B) (Harris et al. 2007). Additionally, expression of Pvf1 was detected in the anal plate primordium (Figure 3B). Importantly, in stage 16 embryos, Pvf1 was absent from the dorsal vessel (Figure 3B). Pvf2 is required for the viability and proliferation of larval hemocytes (Munier et al. 2002). We observed expression of Pvf2 in the proventriculus and the tip of the posterior spiracles of the embryo, whereas Pvf2 could not be detected in the embryonic heart (Figure 3C). Pvf3 is expected to promote embryonic hemocyte migration and cell‐autonomous growth in larval hemocytes (Cho et al. 2002; Sims et al. 2009). Antibody staining against Pvf3 showed no distinct expression pattern in the embryo or in the heart (Figure 3D).

**FIGURE 4:**
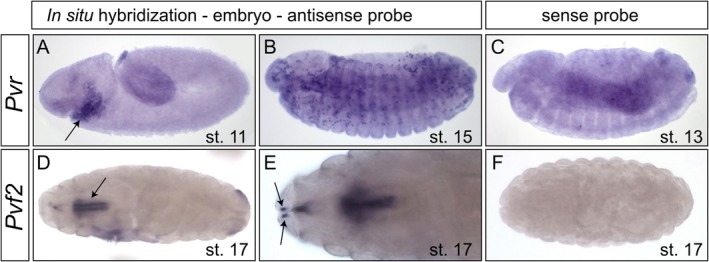
Localization of Pvr and Pvf2 mRNA in the embryo by in situ hybridization. (A) Pvr expression is detected in the head mesoderm of the early embryo, representing the hemocyte pool (Stage 11, arrow). (B) In Stage 15 embryos, the Pvr signal is detectable in hemocytes. (C) Hybridization with the sense probe did not result in a distinct signal above background in Stage 13 embryos. (D, E) Pvf2 mRNA is expressed in the proventriculus (D, arrow) and in the antennal primordia complex (E, arrows). (F) Application of the sense probe did not result in any specific staining.

**FIGURE 5:**
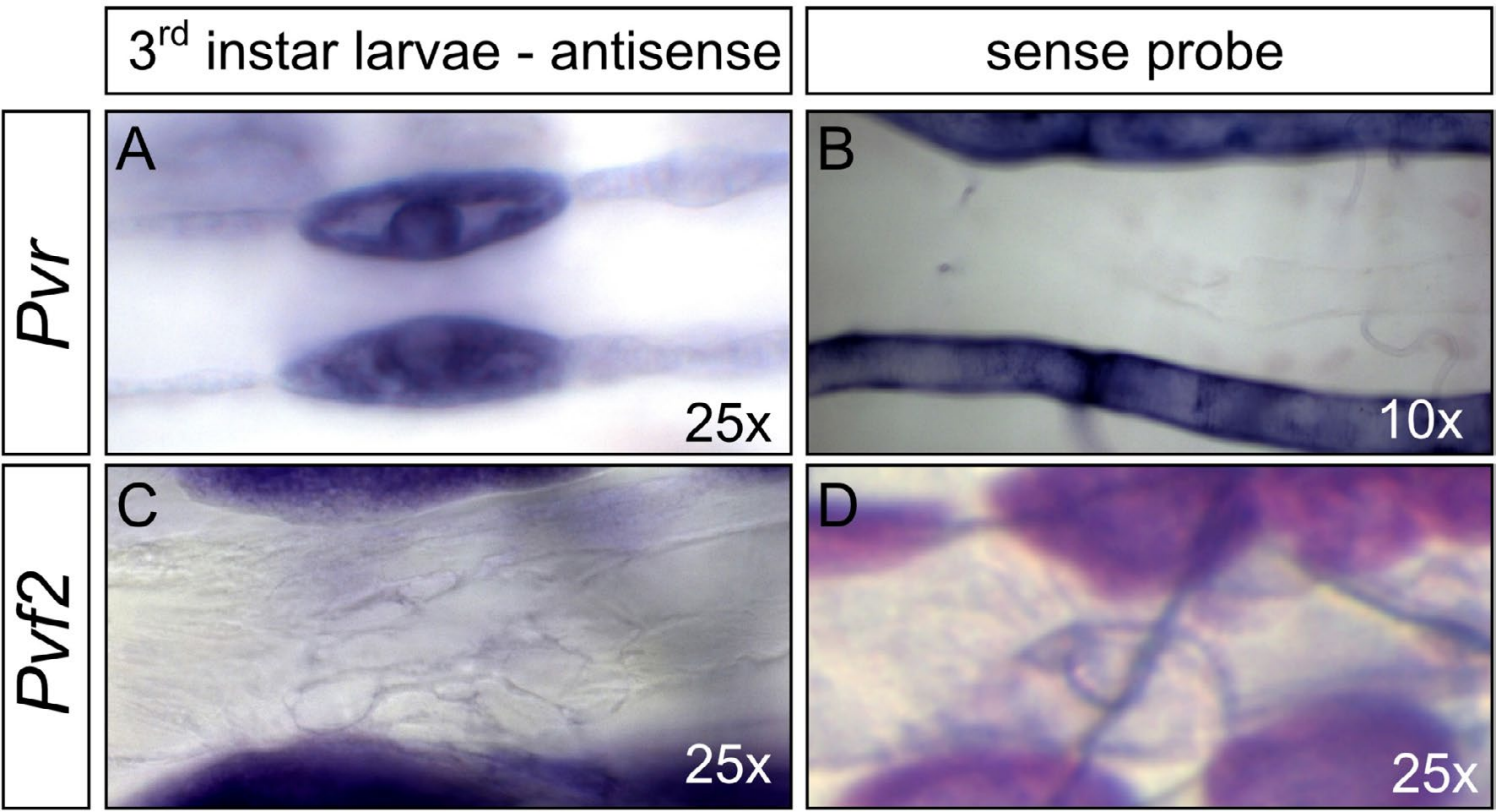
In situ hybridization of *Pvr* and *Pvf2* in third instar larvae. (A) Hybridization of dissected third instar larvae with the *Pvr* antisense probe led to specific staining in valve cells. (B) The sense probes served as a negative control. (C) Distinct expression of *Pvf2* mRNA was not observed in valve cells. (D) The sense probes served as a negative control.

In addition to antibody stainings, we performed in situ hybridization expression studies for Pvr and Pvf2 using stage 11–13 and stage 16–17 embryos (Figure 4) and larvae (Figure 5). We found Pvr expression in the head mesoderm of early embryos (Figure 4A), which is known to be the reservoir region of Pvr‐expressing hemocytes (Figure 4B) (Cho et al. 2002; Heino et al. 2001). In larvae, Pvr was located in cardiovascular valve cells (Figure 5A). Pvf2 mRNA was located in the proventriculus, the posterior spiracles, the primordia of the antenna complex and the head region of embryos (Figure 4D,E). In larvae, no heart‐specific signal could be detected (Figure 5B). In situ hybridizations were repeated several times with varying conditions. Nevertheless, we failed to detect Pvf2 mRNA in larval valve cells, although antibody stainings (see above) clearly indicated the protein's presence in intracellular vesicles. This result may reflect very low mRNA levels at this developmental stage. Notably, Pvf2 is not listed in the FlyAtlas expression data library, indicating low gene expression (Krause et al. 2022). In sum, Pvr transcripts and the protein are present in third instar larval valve cells. The Pvf2 protein is clearly expressed in valve cells; however, we were unable to detect the corresponding mRNA, likely due to sensitivity issues.

### 2.3 RNAi‐Mediated Knockdown of Pvr or Pvf2 Inhibits Valve Cell Differentiation in Third‐Instar Larvae

**FIGURE 6:**
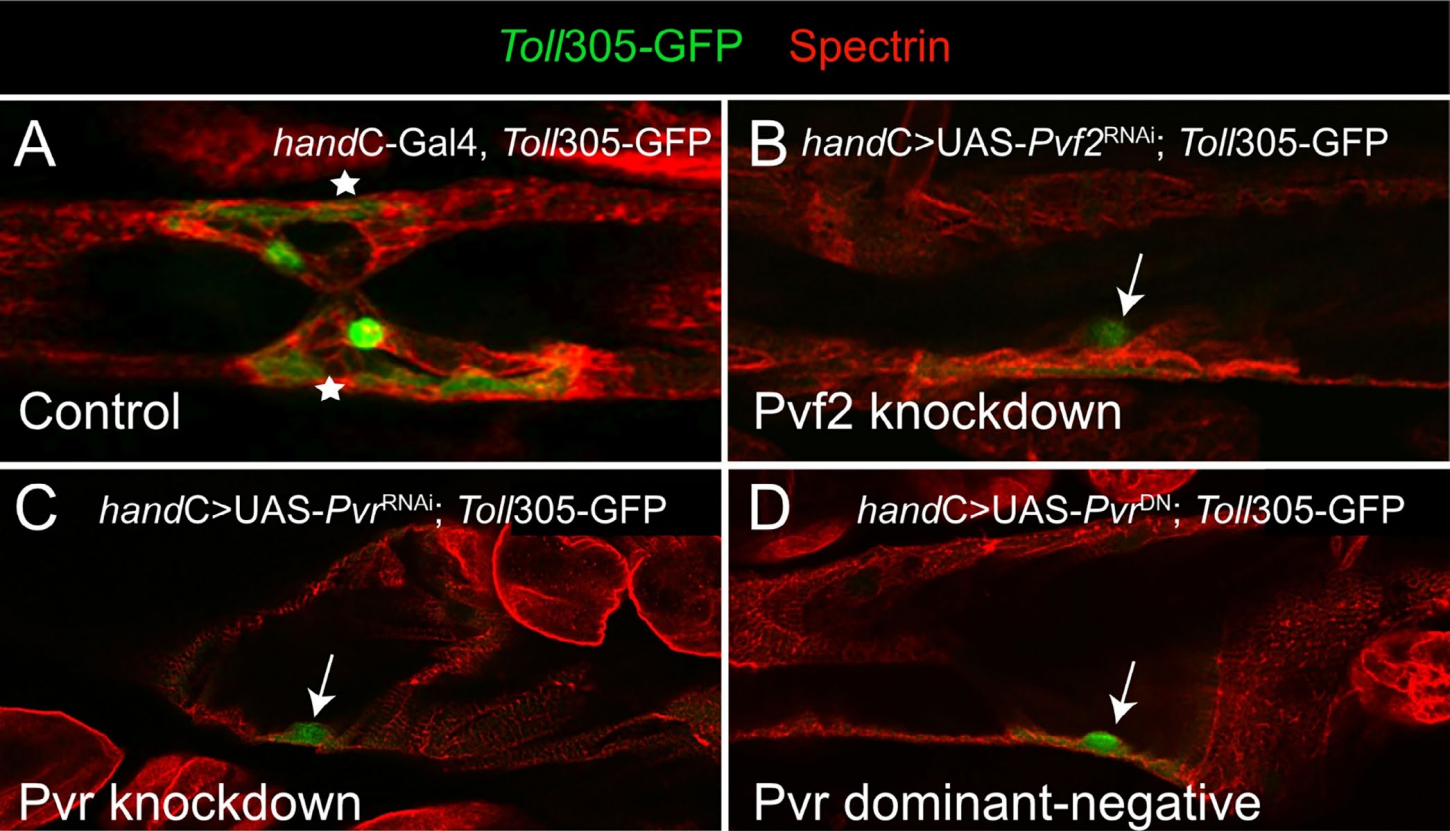
Heart‐specific RNAi‐mediated knockdown experiments of Pvr and Pvf2, and expression of a dominant negative form of Pvr. (A) Wild‐type expression pattern of reporter *Toll*305‐GFP (green) and Spectrin (red) in valve cells of third instar larvae marks the typical cell morphology containing valvosomes and a large cell volume in the heart tube (asterisks). (B–D) Heart‐specific knockdown of Pvr (B), Pvf2 (D), and expression of DN‐Pvr (C) led to an inhibition in valve cell development. Instead, the cells differentiate into cardiomyocytes but retain the GFP signal originating from the *Toll*305‐GFP reporter line (arrows).

Given the specific expression patterns of Pvr and its ligand Pvf2 in cardiovascular valve cells in third instar larvae, the question arises whether Pvr and Pvf2 are necessary for the differentiation process of this cell type. We performed RNAi‐mediated knockdown to reduce Pvr and Pvf2 mRNA levels (Figure 6). Expression of the RNAi constructs was mediated by *hand*‐Gal4, which expresses Gal4 in all cardiac cells from mid‐embryonic stages onward (Paululat and Heinisch 2012; Sellin et al. 2006). In addition, we inhibited Pvr function by expression of a dominant‐negative Pvr variant, UAS‐PvrDN (Rorth 2014) (Figure 6). Heart‐specific knockdown of either the Pvr receptor or its ligand Pvf2 led to an inhibition of cardiovascular valve formation and a lack of fully differentiated valve cells in third‐instar larvae (Figure 6). Because the efficiency of RNAi‐mediated knockdown can vary between individual hairpins, two lines with different RNAi constructs targeting the same gene were analyzed (Table 1). Although the ligands Pvf1 and Pvf3 were not detectable in third instar larval valve cells by antibody staining (Figures 3 and 4), their potential role was nevertheless tested via heart‐specific RNAi‐mediated knockdown. Neither the knockdown of Pvf1 nor of Pvf3 affected larval valve cell development (Table 1), which further corroborates that Pvf2, but not Pvf1 or Pvf3, serves as Pvr ligand in valve cell differentiation.

**TABLE 1:** Genetic constructs used in this study and their individual effects on valve cell differentiation.

Gene or genotype	Construct	Stock identifier	Percentage of animals with valve cell differentiation defects	Ectopic valves
Pvr	UAS‐RNAi	BL13502	50% (*n* = 36)	
Pvr	UAS‐RNAi	BL 105353	30% (*n* = 50)	
Pvr	UAS‐Pvr^dominant‐negative^	Roerth, 2014	45% (*n* = 22)	
Pvf1	UAS‐Pvf1^RNAi^	BL 102699	0% (*n* = 25)	
Pvf1	UAS‐Pvf1^RNAi^	BL 6175	0% (*n* = 26)	
Pvf2	UAS‐Pvf2^RNAi^	BL 102072	29% (*n* = 42)	
Pvf2	UAS‐Pvf2^RNAi^	BL 7629	35% (*n* = 40)	
Pvf3	UAS‐Pvf3^RNAi^	BL 105008	0% (*n* = 21)	
Pvf3	UAS‐Pvf3^RNAi^	BL 37933	0% (*n* = 22)	
Pvr	UAS‐Pvr	Roerth, 2014		0% (*n* = 20)
Pvf2	UAS‐Pvf2	Roerth, 2014		0% (*n* = 20)
Pvr, Pvf2	UAS‐Pvr; UAS‐Pvf2	see above		80% (*n* = 30)
Hand	*handC*‐Gal4; *handC*‐GFP (control and live reporter)	our lab	0% (*n* = 30)	
Hand	*handC*‐Gal4; UAS‐mCD8::GFP (control and live reporter)	our lab	0% (*n* = 30)	
Hand, toll	*handC*‐Gal4; Toll305‐GFP (control and live reporter)	our lab	0% (*n* = 30)	

### 2.4 Overexpression of Pvr and Pvf2 Induces Ectopic Valve Cells

**FIGURE 7:**
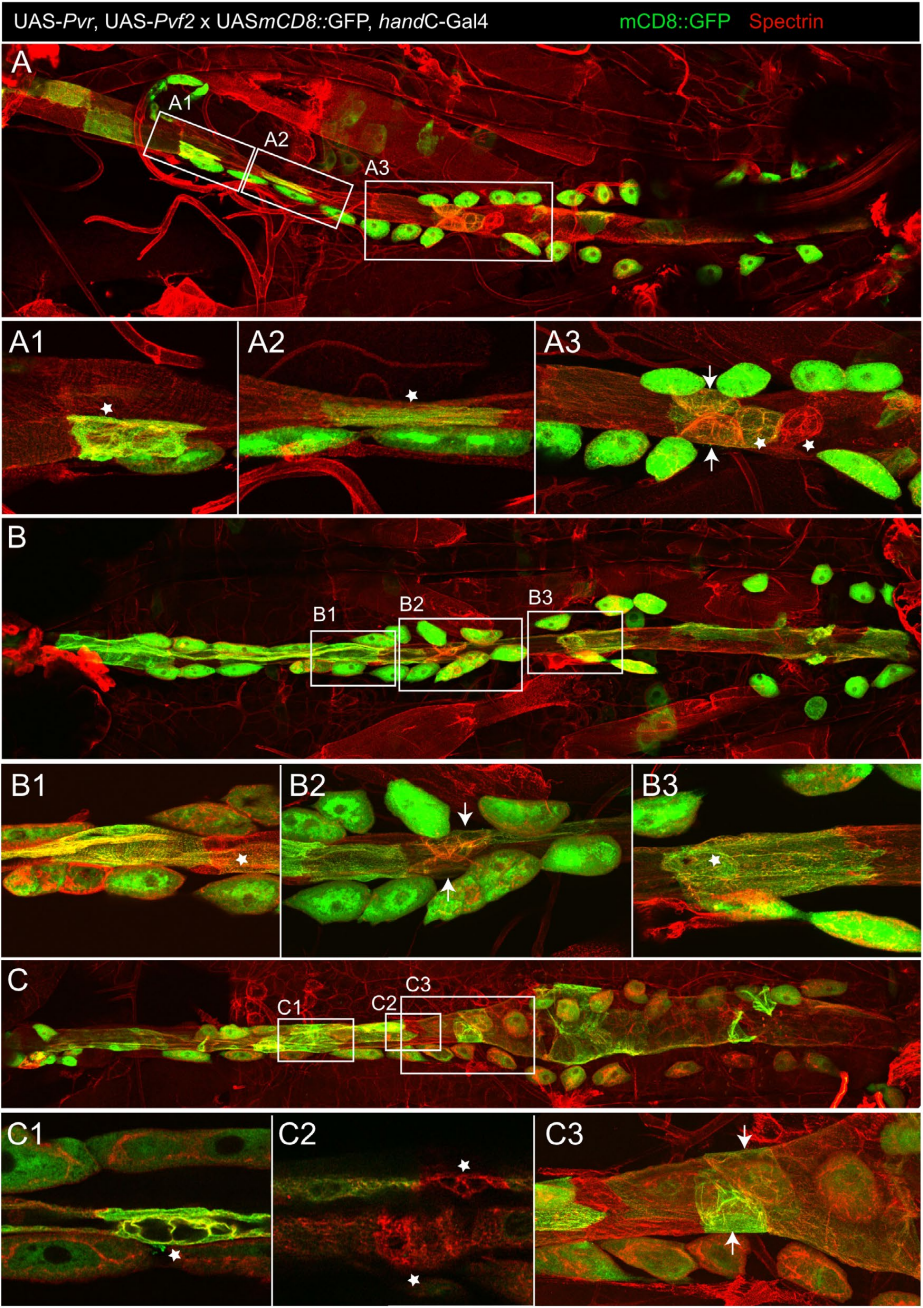
Simultaneous expression of Pvr and Pvf2 causes formation of ectopic valves in third instar larval hearts. (A) Ectopic valves are visualized by an antibody staining against the membrane‐localized marker mCD8::GFP (green) and Spectrin (red). (A1, A2) Two extra valves (asterisks) can be observed in the aortic region and (A3) two extra valve cells are situated in the ventricle (asterisks), adjacent to the wild‐type valve cells (arrows). (B) In this larval heart, two additional valves (asterisks) are observed. (B1) One extra valve (asterisk) is situated in the aorta, (B2) in front of the wild‐type valve cells (arrows), (B3) while one extra valve (asterisk) developed in the ventricle of the heart tube. (C) Overexpression of Pvr and Pvf2 led to three extra valves (asterisks) in the heart tube of this larva. (C1, C2) The extra valves (asterisks) developed in the aorta portion of the heart tube. The wild‐type valve cells are indicated by arrows (C3).

**FIGURE 8:**
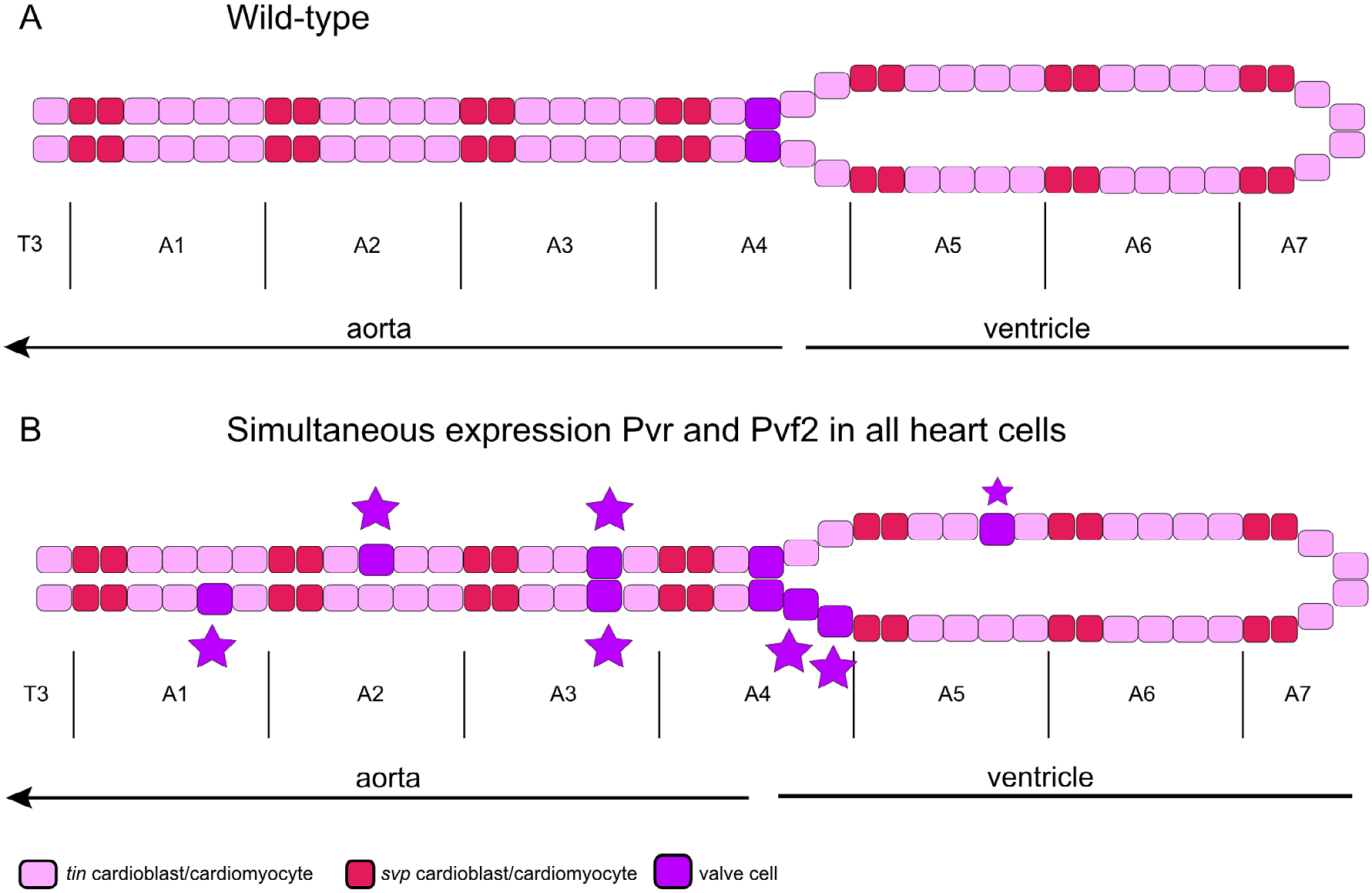
Schematic illustration of a wild‐type heart (A) and a heart, in which simultaneous expression of Pvr and Pvf2 induces the ectopic formation of cardiac valve cells (B). Valve cells are indicated by a purple color, tinman‐expressing cardiomyocytes by pink color, and seven‐up‐expressing ostial cells by red color. Our data indicate that only tinman‐positive cardiomyocytes can be converted. The experimental data underlying this model were generated by the analysis of at least 30 animals.

To analyze if the PDGF/VEGF pathway is not only essential for valve development, but also sufficient for inducing valve cell fate, the receptor Pvr and its ligand Pvf2 were overexpressed in the entire dorsal vessel (Table 1). Heart‐specific overexpression of Pvr or Pvf2 alone did not affect normal differentiation of cardiac valve cells, nor did it induce the expression of ectopic valves elsewhere in the heart tube (Table 1). In contrast, the simultaneous overexpression of the receptor Pvr and the corresponding ligand Pvf2 resulted in randomly situated ectopic valve cell development in the aorta (Figures 7 and 8, Table 1). These extra valves exhibited the typical valve cell morphology, with valvosomes and an enlarged cell volume, as visualized by expression of membrane bound GFP (Figure 7). Previously performed control stainings already confirmed that *hand*C‐Gal4 driven mCD8::GFP has no effect on the formation of valve cells in third instar larval Drosophila hearts (Lammers et al. 2017).

Interestingly, simultaneous ectopic cardiac expression of Pvr and Pvf2 not only led to the differentiation of extra valves in the aorta, but also in the ventricle of third instar larval hearts (Figure 7A3,B3). Additionally, extra valves are positive for the Toll^305^‐GFP reporter signal, indicating proper valve cell differentiation. The Toll305‐enhancer and its expression pattern in the Drosophila heart have been previously described (Lammers et al. 2017; Meyer, Bataille, et al. 2023). The presence of extra valves in the hearts of third instar larvae was observed in 80% of the animals investigated (Table 1).

### 2.5 Transforming Regular Cardiomyocytes Into Valve Cells

A remarkable feature of the *Drosophila* heart is the highly constant number of cells that constitute the heart tube, 104 (Figure 1). Six cardiomyocytes of the heart tube are situated in a hemisegment, four of them express Tinman (known as Tinman‐cardiomyocytes), and two of them express Seven‐up and differentiate into ostia cells of the larval or adult heart, as depicted in Figure 1. A brief overview of the different cell types of the heart is available elsewhere (Lehmacher et al. 2012; Rotstein and Paululat 2016). Upon simultaneous Pvr and Pvf2 expression using *hand*‐Gal4, which drives expression in all cardiomyocytes (Paululat and Heinisch 2012; Sellin et al. 2006), we noted by position that only the Tinman‐positive cardiomyocytes, but never the Seven‐up expressing ostia precursors, are induced to differentiate into valve cells (Figure 8).

In sum, our experiments showed that the PDGF/VEGF pathway is necessary and sufficient to induce valve cell development in distinct cardiomyocytes of the third instar larval heart tube.

## 3 Discussion

The *Drosophila* heart develops in the second half of embryonic development and is formed from a constant number of cardiomyocytes, 104 in total. The cells are arranged in two rows (2 × 52 cells), with two opposing cells that continuously form a lumen. The specification of heart cells and lumen formation during embryonic development has been described on numerous occasions in reviews (Ahmad 2017; Lo et al. 2002; Vogler and Bodmer 2015; Volk et al. 2014) and elsewhere. During larval development, a single cardiac valve composed of two individual cells develops (Lammers et al. 2017). The valve cells differentiate from cardiomyocytes, and the functional valve separates the posterior large‐lumen heart chamber from the narrow‐lumen anterior aorta. Heart valve cells are highly specialized cells with a unique histology. Invaginations of the cell membrane and continuous fusion of special recycling endosomes create large intracellular vacuoles, which give cardiac valve cells a flexible, rounded shape (Meyer et al. 2022). The larval cardiac valve always develops in the same position in every animal, so that 38 consecutive cardiomyocytes always form the ventricle. It has already been shown that the anterior–posterior axis of the heart tube is determined by the activity of Hox genes (Jagla et al. 2001; LaBeau et al. 2009; Lo and Frasch 2003; Lo et al. 2002; Lovato et al. 2002; Monier et al. 2007; Perrin et al. 2004; Ryan et al. 2005). The position of the developing heart valve lies at the boundary between the anterior Ubx and a posterior Abdominal‐A (Abd‐A) expression domain. Thus, although entirely speculative, the position of the heart valve could be determined by Hox genes. However, so far, we have no experimental evidence for this.

The signaling pathway responsible for the differentiation of valve cells remained unknown for a long time. The first important observation can be traced to the work of Zeitouni and colleagues, who specifically sought signaling pathways that regulate the formation of the adult heart in *Drosophila* (Zeitouni et al. 2007). They observed that expression of the PGDF receptor (Pvr) and its ligand, Pvf2, is upregulated during metamorphosis. Inhibition of Pvr function by overexpression of the PvrDN variant partially inhibits valve formation, while ectopic expression of constitutive Pvr (Pvr^lambda^) induces ectopic valve formation. However, the ectopically induced valve cells are always in the direct neighborhood of the canonical ones.

We focused herein on the formation of the single pair of valve cells present in the heart of a third instar larva. We found that both the Pvr receptor and its ligand Pvf2 are expressed in valve cells (Figures 2, 3, 4). This strongly supports an autocrine signaling mechanism, in which the differentiating valve cells produce and secrete Pvf2, which is then received by the Pvr receptor at the plasma membrane of the same cell. Such a precise regulation ensures that only one valve at a distinct position develops, which is highly important for an optimized hemolymph flow in the heart (Meyer and Paululat 2025). Downregulation of either Pvr or Pvf2 inhibits larval valve formation, indicating that both are crucial for initiating valve cell differentiation. However, when we co‐expressed the receptor and ligand in all cardiomyocytes, we observed ectopic valve cells in 80% of animals (see Table 1), both in the aorta and in the ventricular region. Of note, the expression of Pvr or Pvf2 alone has no effect, confirming the interdependence of the two factors. While this is not surprising for ligand expression, given the absence of a receptor that could promote cell differentiation, it is unexpected for receptor expression. At least in the vicinity of the endogenous heart valve cell, one would expect sufficient concentrations of the secreted ligand Pvf2 to be present for ectopic heart valves to develop there. However, we do not see this (Table 1), indicating a highly confined area of activity for Pvf2.

Interestingly, we observed that when Pvr and Pvf2 are co‐expressed, ectopic heart valve cells arise only from the known Tinman‐positive cardiomyocytes, but not from ostia‐forming cardiomyocytes (Figure 8). We suspect that the ostia cells are already specified and therefore cannot be reprogrammed with respect to their developmental fate. A decisive factor for ostium differentiation is the orphan receptor Seven‐up. As previously shown, Seven‐up is activated very early in development and specifies the ostia in the larval and adult heart (Bodmer and Frasch 1999; Gajewski et al. 2000; Mlodzik et al. 1990; Molina and Cripps 2001; Ward and Skeath 2000). Thus, an ostial cell fate apparently excludes valve cell differentiation.

In summary, we extended previous observations on the formation of intracardiac valve cells. Our experiments showed that the PDGF/VEGF pathway is necessary and sufficient for inducing cardiovascular valve development in the heart tube of third instar larvae.

## 4 Materials and Methods

### 4.1 Fly Stocks and Genetics

The following fly stocks were used in this study: *handC*‐Gal4 (Sellin et al. 2006) and *Toll* ^*305*^‐Gal4 from our laboratory. UAS‐Pvr^DN^. UAS‐Pvr, UAS‐Pvf2, UAS‐Pvr(II), UAS‐Pvf2(III) were a gift from Pernille Rorth (Duchek et al. 2001). UASmCD8::GFP (BL5137) and *handC*‐Gal4 were recombined in our laboratory. *Toll305*‐GFP was a gift from Robert Schulz (Wang et al. 2005). RNAi‐lines obtained from the Bloomington *Drosophila* Stock Center (BDSC) and Vienna *Drosophila* Resource Center (VDRC): UAS‐Pvf1‐RNAi (VDRC_102699, VDRC_6175), UAS‐Pvf2‐RNAi (VDRC_102072, VDRC_7629), UAS‐Pvf3‐RNAi (VDRC_105,008, VDRC_37933), UAS‐Pvr‐RNAi (VDRC_13502, VDRC_105353).

### 4.2 Crossings and Genotypes of the Analyzed Flies

All fly lines listed in table1 are homozygous viable. In all images shown, we crossed homozygous Gal4 drivers with homozygous UAS‐effectors and analyzed the F1 progeny. As control, we stained the homozygous Gal4 drivers. Previous work confirmed that there is no obvious difference between hetero‐ or homozygous drivers with respect to valve cell formation (Lammers et al. 2017, and unpublished results).

### 4.3 Antibodies

Anti‐Pvr (made in rat) and Anti‐Pvf2 (made in rat) were a gift from Benny Shilo (Rosin et al. 2004). Anti‐Pvf1 and Anti‐Pvf3 were gifts from Benny Shilo and Pernille Roerth (Duchek et al. 2001). Anti‐Spectrin (made in mouse) was obtained from DSHB and used in a 1:25 dilution.

### 4.4 Immunohistochemistry

Third‐instar larvae were dissected in PBS and fixed in 4% paraformaldehyde (PFA) in PBS for 1 h at RT. After three 10‐min washing steps, specimens were permeabilised with 1% Triton X‐100 in PBS for 1 h at RT, followed by three further washing steps with BBT (0.1% BSA and 0.1% Tween‐20 in PBS) for 10 min each. Subsequently, specimens were incubated for 60 min in a blocking solution containing 1% BSA and 0.1% Tween‐20 in PBS followed by incubation with the primary antibodies (rat anti‐Pvr, 1:500, rat anti‐Pvf1, ‐Pvf2, and ‐Pvf3, 1:100–500) in BBT overnight at 8°C. Samples were rinsed three times with BBT (10 min, RT) and incubated with secondary antibodies (anti‐rabbit Alexa Fluor 488, 1:200, Jackson ImmunoResearch Laboratories, West Grove, PA, USA, RRID:AB_2338680; anti‐mouse Cy3, 1:200, Jackson ImmunoResearch Laboratories, RRID: AB_2338680; anti‐chicken Alexa Fluor 488, 1:200, Jackson ImmunoResearch Laboratories, RRID:AB_2340375) in BBT for 2 h at RT followed by three washing steps with BBT. Samples were embedded in Fluoromount‐G mounting medium containing DAPI (Thermo Fisher, Waltham, MA, USA). Confocal images were acquired using a laser scanning microscope (LSM800 or Pascal5, Zeiss, Jena, Germany) equipped with a Zeiss EC Plan‐Neofluar ×40/NA 1.30 Oil DIC M27 objective, a Multikali PMT detector, and Zen2.6 software. Filters for Alexa Fluor 488, Cy3 and DAPI were used. Image processing was done with Fiji (RRID:SCR_002285) (Rueden et al. 2017; Schindelin et al. 2012) and Affinity Photo (Serif, Nottingham, United Kingdom, RRID:SCR_016952).

### 4.5 In Situ Hybridization for Pvr and pvf2‐Transcripts

To generate RNA probes for in situ hybridization, we used full‐length cDNA clones obtained from BDGP (Berkeley). For Pvr, cDNA clone RE12971, cloned into pFLC‐1 (insert: 4935 bp), was used. For Pvf2, cDNA clone RH402111, cloned into pFLC‐1 (insert: 2119 bp), was used. Sense and anti‐sense RNA probes were synthesized using the “DIG RNA labeling kit” (Roche, Basel, Switzerland). In situ hybridization and double labelling with antibodies were performed as described previously (Kölsch and Paululat 2002).

### 4.6 Transmission Electron Microscopy

Specimens were prepared as previously described (Lehmacher et al. 2012; Meyer et al. 2022; Psathaki et al. 2018; Psathaki and Paululat 2022).

## Funding

This work was supported by grants from the Deutsche Forschungsgemeinschaft (PA 517/13‐1, PA 517/13‐2; SFB1557‐TP12; SFB1557‐Z‐Project; PA517/12‐1, PA517/12‐2 [A.P.]; and HA 6421/4‐1 [H.M.]), the Deutscher Akademischer Austauschdienst (DAAD), and the State of Lower Saxony (ZN2832) to A.P.

## Data Availability

The data that support the findings of this study are available from the corresponding author upon reasonable request.
